# In vivo generation and regeneration of β cells in zebrafish

**DOI:** 10.1186/s13619-020-00052-6

**Published:** 2020-07-02

**Authors:** Bingyuan Yang, Brittney A. Covington, Wenbiao Chen

**Affiliations:** Department of Molecular Physiology & Biophysics, Vanderbilt University School of Medicine, 2215 Garland Avenue, Nashville, TN 37232 USA

## Abstract

The pathological feature of diabetes, hyperglycemia, is a result of an inadequate number and/or function of insulin producing β cells. Replenishing functional β cells is a strategy to cure the disease. Although β-cell regeneration occurs in animal models under certain conditions, human β cells are refractory to proliferation. A better understanding of both the positive and the negative regulatory mechanisms of β-cell regeneration in animal models is essential to develop novel strategies capable of inducing functional β cells in patients. Zebrafish are an attractive model system for studying β-cell regeneration due to the ease to which genetic and chemical-genetic approaches can be used as well as their high regenerative capacity. Here, we highlight the current state of β-cell regeneration studies in zebrafish with an emphasis on cell signaling mechanisms.

## Background

An absolute or relative deficiency of functional insulin producing β cells is the pathological feature of both types of diabetes (Weir et al., 1990). Although the disease conditions can be managed by a number of drugs including insulin, insulin sensitizers, and glucose reabsorption inhibitors, current treatments are insufficient to prevent diabetic complications and can cause side effects, even when closely followed (Pothineni & M. J., 2015; Corathers et al., 2013). Restoring functional β-cell mass may cure both type 1 and type 2 diabetes. Indeed, transplanting cadaveric islets gives recipients several years of insulin independence (Shapiro, 2000). The scarcity of compatible cadaveric donors and lifelong immune suppression limit its broad application. A heavily investigated alternative source is β cells derived from human embryonic stem cells or induced pluripotent stem cells (Rezania et al., 2012; Pagliuca et al., 2014; Benthuysen et al., 2016). Despite tremendous progress, these β-like cells are still inferior to β cells from donors (Tremmel et al., 2019). Even if fully functional β cells can be generated in mass quantities, their preservation after transplantation may still require immunosuppression. An alternative to in vitro β-cell production is induction of endogenous regeneration (Aguayo-Mazzucato & Bonner-Weir, 2018). Unlike in vitro generated β-like cells, in vivo generated β cells situate in their natural environment, integrate into the intricate paracrine regulatory network in the islet, and deliver insulin directly to the portal vein. As such, they will likely function better. Recent studies in animal models suggest that in vivo β-cell regeneration is a viable approach to replenish β-cell mass in diabetic models (Aguayo-Mazzucato & Bonner-Weir, 2018).

Pancreatic β-cell regeneration occurs physiologically in conditions of increased insulin demand such as pregnancy (Toselli et al., 2014; Kim et al., 2010; Karnik et al., 2007; Parsons et al., 1992) and obesity (Yamamoto et al., 2017; Bonner-Weir, 2000; Liu et al., 2017). Regeneration also occurs in experimentally induced conditions of insufficient insulin function, such as partial pancreatectomy (Togashi et al., 2014; Noèlia & Eduard, 2014), β-cell ablation (Cheng et al., 2015; Thorel et al., 2010), and insulin receptor antagonist treatment (Jiao et al., 2014). Three general mechanisms of in vivo β-cell regeneration have been reported in animal models: self-replication or proliferation, neogenesis or progenitor differentiation, and transdifferentiation (Aguayo-Mazzucato & Bonner-Weir, 2018). Proliferation refers to the generation of new β cells from existing ones by cell division. It is the predominant mode of β-cell expansion from late gastrulation to adulthood in rodents (Dor et al., 2004; Teta et al., 2007). Neogenesis refers to the generation of β cells from endocrine progenitors. This occurs during development as well as in adults (Bonner-Weir et al., 2012; Huising et al., 2018). Transdifferentiation refers to β-cell production from differentiated non-β cells, usually from a cell type of related lineage such as pancreatic endocrine cells, hepatocytes, and intestinal endocrine cells. It occurs in certain conditions such as severe β-cell depletion and under some drug treatments (Thorel et al., 2010; Chera et al., 2014; Lee et al., 2018). Although evidence for all 3 mechanisms of β-cell regeneration exists (Bonner-Weir et al., 2010; Inada et al., 2008; Bouwens et al., 1994), it is generally believed that proliferation is the predominant mechanism (Dor et al., 2004; Teta et al., 2007). However, with advance of age, the capacity of β-cell proliferation and regeneration rapidly declines (Perl et al., 2010; Chen et al., 2011; Swenne, 1983). A recent finding revealed that the decline is accompanied by an increase of DNA methylation in β cells (Avrahami et al., 2015).

Compared to rodents, adult human β cells are resistant to proliferation. Once reaching a peak by early adulthood, human β-cell mass remains steady with very slow turnover (Butler et al., 2003; Gregg et al., 2012; Kassem et al., 2000). However, evidence of adult islet plasticity in response to insulin resistance exists (Mezza et al., 2019). Attempts to enhance human β-cell proliferation have been hindered by inadequate knowledge of the signaling pathways that promote cell cycle progression and prevent cell cycle reentry (Bernal-Mizrachi et al., 2014; Kulkarni et al., 2012; Stewart et al., 2015). What causes the resistance of human β-cell proliferation is not known. Thus, it is of paramount importance to identify signaling pathways that can specifically activate β-cell proliferation as well as pathways that confer its mitotic resistance for developing treatment targeting these pathways.

Zebrafish have been extensively used for understanding vertebrate biology and human diseases. This is due to many experimental advantages the model possesses. It is highly tractable genetically because of fecundity and oviparous reproduction. Highly efficient mutagenesis and transgenesis can be achieved with minimal training and commonly available reagents and equipment. As such, a large number of mutant and transgenic lines are available at nominal cost from various resource centers. Rapid development of translucent embryos makes zebrafish a favorable model organism for research, as many developmental events can be directly visualized. The small size of the embryo/larvae makes them compatible with chemical screening as they can be housed in microtiter plates for treatment and the anatomical or behavioral results can be easily observed. In addition to developmental biology, zebrafish have been increasingly used for modeling human diseases for mechanistic investigations and drug development (Lam Pui-Ying, 2019).

**Fig. 1:**
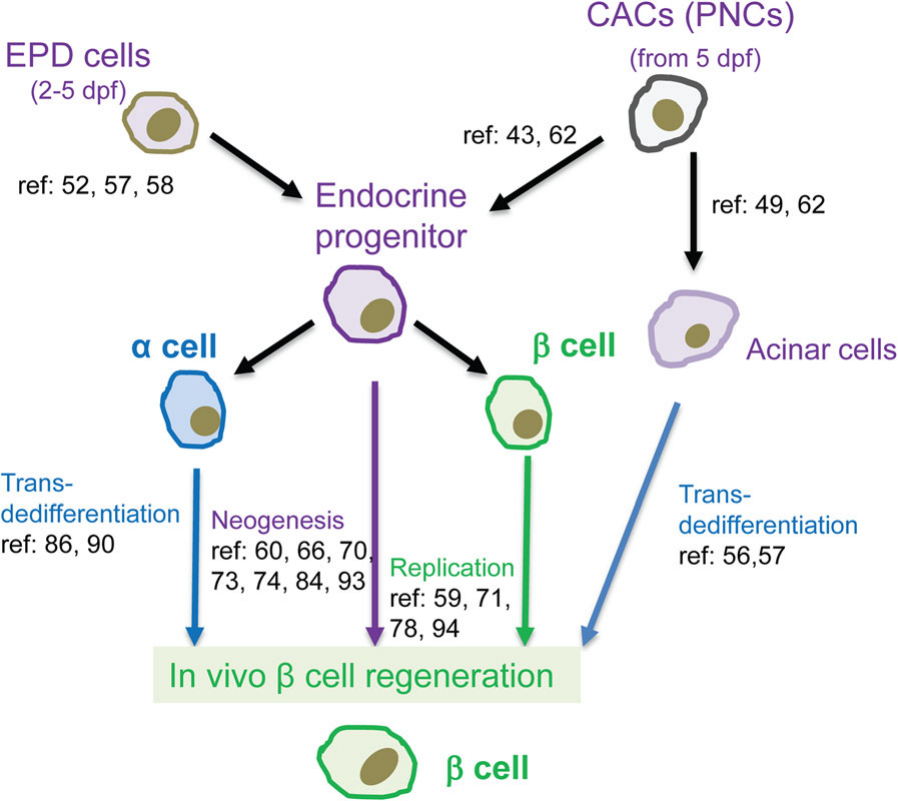
Cell sources for in vivo generation of β cells in zebrafish. Cells in the extrapancreatic duct linking the pancreas to the gut (EPD) differentiate into endocrine progenitor cells that form the ventrally derived β cells in the principal islet. Centroacinar cells (CACs) are a specialized ductal pancreatic cell type that differentiates into progenitors of acinar, ductal, and endocrine cells. Under certain conditions, new β cells can be generated through transdifferentiation, neogenesis and proliferation

The zebrafish pancreas exhibits a remarkable capacity for regeneration (Delaspre et al., 2015; Ghaye et al., 2015; Moss et al., 2009). Recent findings in zebrafish β-cell regeneration have helped us understanding signaling pathways and transcription factors involved in β-cell neogenesis, transdedifferentiation and proliferation (Fig. 1). We will review these studies based on the methods employed to trigger β-cell formation (Table 1).

**Table 1:** Conditions that promote β-cell regeneration

	Inducer	Effects	Developmental stage	Cell source	Related mechanism	Modifer Screen	Gene/target identified	Related pathway	Reference
Manipulation of Insulin signaling	Transgenic expression of a dominant-negative IGF1R	Insulin resistance	Embrynonic, Larval and adult	Progenitors and β cells	Proliferation	None	igf1r	Insulin signalling	(Maddison et al., 2015)
	Knockout of insra/insrb	Insulin resistance	Embryonic and larval	Unknown	N.A.	None	insra/insrb	Insulin signalling	(Yang et al., 2017)
	Transgenic expression of a dominant-negative IRS2	Insulin resistance	Embryonic	Progenitors	Neogenesis	None	irs2	Insulin signalling	(Ye et al., 2016)
	Nutrient	Increased insulin demand	larval	Progenitors	Neogenesis	Candidate drugs	fgf1/rapamycin	FGF, mTORC1	(Maddison & Chen, 2012; Li et al., 2014)
	Nutrient	Increased insulin demand	Larval	Progenitors and β cells	Neogenesis	Candidate drugs	g secretase inhibitor and rapamycin	Notch, mTORC1	(Ninov et al., 2013)
	β-cell ablation	Decreased insulin supply	Larval	Progenitors and β cells	Neogenesis	Chemical library	Adenosine Receptor A2aa (agonist)	Adenosinergic	(Andersson et al., 2012; Schulz et al., 2016)
	β-cell ablation	Decreased insulin supply	Larval	β cells	Proliferation	Chemical library	TBK1/IKKε (inhibitor)	PKA, mTORC1	(Xu et al., 2018)
	β-cell ablation	Decreased insulin supply	Larval	Progenitors	Neogenesis	Mutation	Sox9b	Retinoic acid signaling; Notch signaling	(Manfroid et al., 2012; Huang et al., 2016)
	β-cell ablation	Decreased insulin supply	Larval	Progenitors	Neogenesis	Mutation	Dnmt1	N.A.	(Anderson et al., 2009)
	β-cell ablation	Decreased insulin supply	Larval	Progenitors	Neogenesis	Mutation	fhl1b	N.A.	(Xu et al., 2016)
	β-cell ablation	Decreased insulin supply	Larval	α cells	Trandedifferentiation	Morpholino, antibody	gcg	non-gluconeogenic effects of glucagon	(Ye et al., 2015)
	β-cell ablation	Decreased insulin supply	Larval	α cells	Trandedifferentiation	Mutation	Igfbp1	IGF1 signaling	(Lu et al., 2016)
Chemical genetics	Chemicals	Inhibition or activation of drug targets	Larval	Progenitors	Neogenesis	None	ALDH1A1 (inhibitor), IMPDH (inhibitor)	RA, Nucleotide synthesis	(Rovira et al., 2011; Shen et al., 2013)
	Chemicals	Inhibition or activation of drug targets	Larval	Progenitors and β cells	Proliferation/ Neogenesis	None	NFkb activators, Serotonergic activators	NFkB, Serotonergic, glucorcorticoid analogs	(Wang et al., 2015; Janjuha et al., 2018)
	Chemicals	Inhibition or activation of drug targets	Larval	β cells	Proliferation	None	RXR agonists, serotonin boosters, GR agonists	RA, Serotonergic, glucocorticoid	(Tsuji et al., 2014)
	Notch inhibition	Precocious β-cell differentiation	Larval	Progenitors	Neogenesis	Chemical library	CDK5 (inhibitor)	N.A.	(Liu et al., 2018)
Other	Microbiota	Unknown	Larval	Progenitors	Proliferation	larvae stage	BefA	N.A.	(Hill et al., 2016)
	CDK4	Cell division activation	Larval	β cells	Proliferation	larvae stage	Cyclin-Dependent Kinase 4	N.A.	(Li et al., 2013)
	Genetic Lineage Tracing	Suppression of target gene	embryonic development	Acinar	Trandedifferentiation	None	Ptf1α	N.A.	(Hesselson et al., 2011; Dong et al., 2008)

## A primer on zebrafish β-cell development

The zebrafish and mammalian pancreas show striking similarities in the molecular control of development, cellular and subcellular architecture, and physiological function (Pauls et al., 2007; Pack et al., 1996; Kinkel & Prince, 2009; Parsons et al., 2009). Like mammals, the zebrafish pancreas derives from the dorsal and ventral buds of *pdx1*-expressing foregut endoderm cells. The principal islet is formed at 24 h post-fertilization (hpf) by the coalescence of scattered endocrine cells from the first wave of differentiation in the dorsal bud that emerges before 18 hpf (Wang et al., 2011; Biemar et al., 2001; Kimmel et al., 2011). The first wave of β cells are mitotically quiescent and functionally immature (Hesselson et al., 2009). The principle islet undergoes further expansion with endocrine cells from the second wave of differentiation starting at 40 hpf from the ventral bud-derived cells (Hesselson et al., 2009), which also gives rise to acinar and ductal cells (Field et al., 2003). However, suppression of Ptf1a activity during pancreatic development induces acinar to endocrine conversion (Hesselson et al., 2011; Dong et al., 2008). The principal islet continues to expand with endocrine cells differentiated from the FGF-responsive extrapancreatic duct (EPD) at the head of the pancreas between 2 to 5 days post-fertilization (dpf) (Kimmel et al., 2011; Chung et al., 2010; Dong et al., 2007), and secondary islets form after 5 dpf from endocrine progenitors derived from the pancreatic Notch-responsive cells (PNCs) in the intrapancreatic ductal cells (IPDs) as the pancreas expands caudally (Parsons et al., 2009; Wang et al., 2011), as well as from β-cell proliferation (Ninov et al., 2013; Tsuji et al., 2014). Whether EPD continues to contribute to principal islet expansion is unknown. Inhibition of Notch signaling causes precocious endocrine differentiation and secondary islet formation (Parsons et al., 2009). Modulating the duration and/or extent of Notch signaling inhibition can uncouple amplification and differentiation of the progenitor (Ninov et al., 2012). In addition to endocrine progenitors, these specialized ductal PNCs are also the source of other ductal cells, acinar cells, and centroacinar cells (CACs) in adults, and are morphologically similar to CACs (Delaspre et al., 2015). CACs are situated at the tips of the pancreatic duct inside the acinus and contribute to endocrine cell regeneration after β-cell ablation or partial pancreatectomy (Wang et al., 2011; Beer et al., 2016).

It is believed that β-cell proliferation is the predominant mechanism for expanding adult β cells in mice (Dor et al., 2004; Stamateris et al., 2013; Dor, 2006). However, in zebrafish larvae, β-cell proliferation is not predominant and is dependent on developmental stages and physiological states (Singh et al., 2017). Most advantages of zebrafish over other vertebrate models only hold true in larvae. Early larval zebrafish have only one (principal) islet. This allows rapid analysis of cell number changes. Combined with other already discussed advantages, zebrafish have allowed several groups to identify small molecule modulators and molecular mechanisms of β-cell regeneration (Andersson et al., 2012; Wang et al., 2015; Moon et al., 2020). Nevertheless, the compounds identified in zebrafish may provide new therapies and help identify new therapeutic targets for β-cell regeneration therapy in patients. Here, we highlight some of these studies according to the stimulus of β-cell generation. We emphasize the signaling mechanisms leading to β-cell formation in these models, which provide a potential avenue for the development of anti-diabetic drugs through in vivo β-cell regeneration.

## β-Cell induction by physiological stimuli

### Insulin signaling

The β-cell deficit of Type 2 diabetics may be due to insufficient β-cell production during development and/or defective compensatory β-cell production in response to insulin resistance (Costes et al., 2013). Therefore, understanding physiological β-cell generation is critical for comprehension, prediction, and possibly prevention of diabetes susceptibility.

A key regulator of β-cell number is insulin signaling. Insufficient insulin signaling increases both β-cell number and function to maintain glucose homeostasis (Maddison et al., 2015; Yang et al., 2017; Ye et al., 2016). Defect or breakdown of the compensatory response is the root cause of type 2 diabetes. Although the mechanisms of this compensatory response are not fully delineated, both β-cell extrinsic and intrinsic mechanisms have been proposed (Mezza et al., 2019). The compensatory response is conserved in zebrafish (Yang et al., 2017; Ye et al., 2016). For example, elimination of the insulin receptor by concurrent inactivation of both insulin receptors (*insra* and *insrb*) leads to an increase of β cells (Yang et al., 2017). This compensatory response occurs as early as the first wave of β-cell differentiation. Blocking insulin activity by knocking down *insra*, expressing a dominant-negative IRS2, or exposure to the AKT2 inhibitor from early embryogenesis all cause a significant increase of β cells as early as 24 hpf at the expense of α-cell differentiation (Ye et al., 2016). Similarly, suppressing insulin signaling only in skeletal muscle, a major insulin sensitive tissue, also leads to an increase of β-cell number (Maddison et al., 2015). In this case, the increase occurs later and more gradually, likely due to the tissue restricted insulin resistance. The increase of β cells is at least in part due to proliferation (Maddison et al., 2015).

### Nutrient stimulated β-cell generation

β cells are nutrient sensors. Nutrient ingestion stimulates insulin secretion and increases insulin demand. Two groups have demonstrated that nutrients stimulate β-cell production in larval zebrafish (Maddison & Chen, 2012; Ninov et al., 2013). By culturing 6 or 18 dpf larvae in 5% fresh chicken egg yolk for 8 h, Maddison and Chen showed a rapid increase of β cells by the end of culture. Co-incubation with 5-ethynyl-2′-deoxyuridine (EdU) did not identify proliferating β cells, indicating that the increase is due to neogenesis (Maddison & Chen, 2012). The neogenesis is caused by persistent stimulation of existing β cells since activation of the nutrient-secretion coupling system either pharmacologically or genetically also increased β-cell production (Li et al., 2014). This suggests a non-cell autonomous mechanism in which existing β cells emit a signal to induce differentiation of the yet unidentified responding progenitors, likely the EPD cells, PNCs or its descendants. Using candidate drug screen, the group identified FGF1 as the β-cell signal responsible for induction of β-cell neogenesis (Li et al., 2016). Re-expression of human FGF1 in β cells rescues the defective response in *fgf1−/−* fish. Mechanistically, persistent stimulation of β cells causes mild ER stress, which triggers FGF1 secretion (Li et al., 2016). Ninov and colleagues demonstrated nutrient-dependent β-cell production in older zebrafish larvae (Ninov et al., 2013). At 15 dpf when the larvae were fed with a high calorie diet, more secondary islets formed than in larvae fed with a low-calorie diet. The β cells were generated from NRCs and at least in part from proliferation. Both neogenesis and proliferation require nutrients and can be inhibited by fasting or rapamycin (Ninov et al., 2013), indicating a role for mTORC1 activity. It is likely overnutrition or a high calorie diet also induces neogenesis and proliferation in adults, but such studies have not been reported.

### Microbiota

The gut microbiota has been shown to influence vertebrate development and physiology (Lynch & Pedersen, 2016). Zebrafish gut microbiota is established after hatching and can be suppressed using antibiotics (Rawls et al., 2004). Recently, the Guillemin lab reported an interesting finding that demonstrated a role for the microbiota in early pancreatic β-cell development (Hill et al., 2016). Germ-free zebrafish have fewer β cells than the control. The β-cell number can be normalized by the addition of bacteria that express a conserved previously undescribed secreted protein, BefA (β-Cell Expansion Factor A). Intriguingly, when added in the medium, recombinant BefA, a protein of 261 residues, could restore β-cell number in germ-free larvae to control levels. BefA homologs from the human gut microbiome display similar activity in zebrafish. BefA seems to induce more EdU-positive β cells indicative of proliferation, although EdU was applied for 48 h and the labeling could be from endocrine progenitor cells (Hill et al., 2016). It will be interesting to determine whether the activity requires the entire protein or only a fragment. Equally interesting is the BefA signaling mechanism. For example, it may stimulate β cells directly or cause insulin resistance in other tissues.

## Chemical induced β-cell generation

While nutrient-induced β-cell generation is important for establishing robust β-cell mass before the onset of diabetes, it has limited therapeutic potential. By contrast, drug-induced in vivo β-cell generation has tremendous therapeutic potential. A number of chemical screens to induce β-cell generation in zebrafish have been reported (Andersson et al., 2012; Rovira et al., 2011; Shen et al., 2013). The Parsons lab uses precocious secondary islet formation as the readout for increased β-cell neogenesis (Rovira et al., 2011). By exposing larvae from 2.5 dpf to 5 dpf to more than 3000 compounds, they identified 6 hits. Two of the hits, DSF and MPA, have been approved for the treatment of alcohol abuse and immunosuppression, respectively. Further analysis demonstrated that DSF acts by suppressing retinoic acid synthesis while MPA acts by lowering cellular GTP levels by inhibiting inosine 5′-monophosphate dehydrogenase (IMPDH) (Rovira et al., 2011). The Parsons and Mumm groups developed a high-throughput screen using signal intensity of an insulin promoter driven fluorescent protein in transgenic larvae as the readout (Wang et al., 2015). By exposing the transgenic larvae to individual compounds from 3 to 7 days of age, they identified 24 hits from over 3000 compounds. Some of the compounds induced precocious secondary islets, which suggests they promote neogenesis. Two NF-KB inhibitors are in this group, indicating suppression of NF-KB promotes β-cell differentiation. Other drugs specifically increased β-cell number without accelerating secondary islet formation. Several of these compounds activated the serotonergic signaling pathway and promoted β-cell proliferation (Wang et al., 2015).

Compounds that specifically promote β-cell differentiation are more desirable. It is well established that inhibition of Notch signaling in the Notch-responsive ductal cells promote endocrine progenitor differentiation, resulting in the production of all islet endocrine cell types (Parsons et al., 2009). By co-administering a Notch inhibitor along with more than 2000 compounds from 3 dpf to 5 dpf, Liu et al. identified a CDK5 inhibitor that specifically enhanced β-cell differentiation (Liu et al., 2018). Inhibition of CDK5 also enhanced β-cell differentiation in mouse islet explants, in mice with pancreatic ductal ligation, and in human iPS cells (Liu et al., 2018).

To identify compounds that promote β-cell proliferation directly, the Stainier lab adopted the FUCCI system to mark proliferating β cells (Tsuji et al., 2014). They found that during the first 6 days of development β cells are mitotically quiescent except for two stages, 36 hpf and 144 hpf. By exposing embryos to more than 2000 compounds from 72 to 96 hpf, they identified 20 small molecules that enhance β-cell proliferation during the quiescent period. Of note, most of the compounds affect serotonin, retinoic acid or the glucocorticoids signaling pathway, with the latter two groups also promoting β-cell proliferation after ablation (Tsuji et al., 2014). The serotonergic pathway has been shown to mediate adult β-cell proliferation during pregnancy (Kim et al., 2010) and perinatal β-cell proliferation (Moon et al., 2020) in mice through HTR2B. The identification of the serotonergic pathway from two independent zebrafish screens further validates the relevance of this approach (Wang et al., 2015; Janjuha et al., 2018).

## Ablation-induced β-cell regeneration

Type 1 diabetes results from complete or near complete β-cell loss. In zebrafish, complete or near complete β-cell loss by pancreatectomy or drug-induced killing causes robust β-cell regeneration (Delaspre et al., 2015; Moss et al., 2009; Andersson et al., 2012; Curado et al., 2007). These regenerated β cells are from neogenesis, proliferation, and *α*- to β-cell transdifferentiation. Understanding the molecular mechanisms underlying this response may shed light on ways to induce β-cell regeneration in Type 1 diabetes patients, if autoimmunity can be controlled.

Several genes, *sox9b, dnmt1, gcg* and *fh1b,* have been found to play an important role in ablation-induced β-cell regeneration (Manfroid et al., 2012; Anderson et al., 2009; Ye et al., 2015; Li et al., 2009). Manfroid and colleagues found that ablation-induced β-cell regeneration is severely impaired in *sox9b* mutants. This is because Sox9b is critical for the formation of the PNCs and CACs (Manfroid et al., 2012; Huang et al., 2016). Anderson and colleagues found ablation-induced β-cell regeneration is surprisingly enhanced in *dnmt1* mutant larvae, suggesting decreased DNA methylation promotes β-cell regeneration (Anderson et al., 2009). In mice, near complete β-cell ablation triggers α- or δ- to β-cell transdifferentiation (Thorel et al., 2010; Chera et al., 2014). However, the Anderson group showed that only α cells, but not δ cells, contributed to β-cell regeneration after β-cell ablation in zebrafish (Ye et al., 2015). Using a combination of pharmacological and morpholino antisense oligonucleotide-mediated knockdown approaches, they demonstrated that the α-cell transdfferentiation was dependent on glucagon but independent of gluconeogenesis (Ye et al., 2015). This may explain the findings of Li and colleagues, who demonstrated that persistent killing of β cells by insulin promoter-directed expression of diphtheria toxin A chain also decreased α cells (Li et al., 2009). Lu et al. found that after β-cell ablation, *igfbp1* expression is increased. Igfbp1 could potently promote β-cell regeneration by triggering α- to β-cell transdifferentiation via inhibiting the IGF signaling pathway (Lu et al., 2016). This IGFBP1 function is conserved in mice and human islets (Lu et al., 2016). Another gene that regulates β-cell neogenesis is *fhl1b*^91^. *fhl1b* mutants have enhanced β-cell regeneration due to increased *pdx1* and *neurod* expression in the EPD (Xu et al., 2016).

Chemicals that promote β-cell regeneration after ablation may be exploited for novel drug development. Because pancreatectomy is labor-intensive and incompatible with chemical screens, all chemical screens used metronidazole (MTZ)-mediated ablation of β cells expressing a fluorescent protein fusion of bacterial nitroreductase (NTR) (Curado et al., 2007; Pisharath et al., 2007). Ablation can be done easily by adding MTZ in the medium. The fluorescent protein also provides a marker for the ablation efficiency and a readout of the subsequent regeneration. Using this system, the Stainier group screened more than 7000 compounds and identified NECA as a compound that markedly increases β-cell regeneration (Andersson et al., 2012). It does so by activating adensosine GPCR signaling. Both neogenesis and proliferation contribute to β-cell regeneration. Although this compound only had a limited capacity to induce β-cell proliferation in larval zebrafish, it can potently induce β-cell proliferation in a streptozotocin (STZ)-induced mouse T1D model (Andersson et al., 2012). In addition, pharmacological and genetic disruption of the A2a receptor diminished β-cell proliferation during pregnancy in mice (Schulz et al., 2016). In another screen using a similar system, the Shin lab screened 75 compounds and identified BX795 as a stimulator of β-cell regeneration (Xu et al., 2018). Further analyses indicated that BX795 acts by inhibiting TBK1/IKKε. This finding led to the identification of an even more potent compound known to inhibit TBK1/IKKε, PIAA. PIAA primarily increased β-cell proliferation in zebrafish and a STZ-induced mouse T1D model. Further mechanistic studies revealed that PIAA inhibits TBK1/IKKε phosphorylation of PDE3, resulting in activation of PKA and its target mTORC1, leading to β-cell specific proliferation (Xu et al., 2018).

## Factors that limit β-cell proliferation

Like in mammals, β-cell proliferation in zebrafish also declines with age (Janjuha et al., 2018). Comparing β cells from younger and older zebrafish, aging islets exhibit signs of chronic inflammation (Janjuha et al., 2018). Further investigation indicates that β cells with high NF-kB signaling proliferate significantly less compared to their neighbors with low activity. The cells with active NF-kB signaling also exhibit premature upregulation of socs2, an age-related gene that inhibits β-cell proliferation (Janjuha et al., 2018). Another potential limitation of β-cell replication is insufficient activity of cyclin-dependent kinase 4 (CDK4). This may be due to age-dependent increased expression of cyclin-dependent kinase inhibitors in β cells, such as p27 (Georgia & Bhushan, 2006), p21 (Fatrai et al., 2006), and p16 (Helman et al., 2016), as observed in mice. By ectopically expressing in β cells a mutant CDK4 (CDK4^R24C^) that is insensitive to inhibition by cyclin-dependent kinase inhibitors, β-cell number could be increased through enhanced proliferation (Li et al., 2013).

## Conclusion

Replenishment of new glucose-responsive β cells is the best therapeutic approach to cure diabetes. Here, we summarized the recent advances in β-cell regeneration in vivo using the zebrafish model. Distinguishing the cell sources for in vivo generation of β cells and understanding the mechanisms underlying these processes are of great importance for designing strategies to achieve a β-cell based cure.

## Perspective

In vivo β-cell regeneration is a potential cure for both types of diabetes. However, there is still much that needs to be done before this strategy can be implemented. Continued drug screening efforts in zebrafish will likely contribute to its eventual success. In addition to screening larger compound libraries, future zebrafish studies should also address the toxicity and specificity of the identified compounds. Translation to humans should remain the focus. For any given compound that promotes β-cell regeneration in zebrafish, its efficacy and specificity should be assessed in rodents as some of the previous studies have done (Schulz et al., 2016; El Ouaamari et al., 2016). Except for these that promote neogenesis, the compounds should also be tested on human islets whenever possible. Since multiple pathways may be involved in limiting proliferation of, or transdifferentiation to, human β cells, combined treatment of multiple compounds may be productive. Positive human islet data should pave the way for clinical trials and subsequent drug approval.
